# Value of TIRADS, BSRTC and FNA-BRAF^V600E^ mutation analysis in differentiating high-risk thyroid nodules

**DOI:** 10.1038/srep16927

**Published:** 2015-11-24

**Authors:** Yu-zhi Zhang, Ting Xu, Dai Cui, Xiao Li, Qing Yao, Hai-yan Gong, Xiao-yun Liu, Huan-huan Chen, Lin Jiang, Xin-hua Ye, Zhi-hong Zhang, Mei-ping Shen, Yu Duan, Tao Yang, Xiao-hong Wu

**Affiliations:** 1 Department of Endocrinology, the First Affiliated Hospital with Nanjing Medical University, 300 Guangzhou Road, 210029, Nanjing, China; 2 Department of Pathology, the First Affiliated Hospital with Nanjing Medical University, 300 Guangzhou Road, 210029, Nanjing, China; 3 Department of Ultrasound, the First Affiliated Hospital with Nanjing Medical University, 300 Guangzhou Road, 210029, Nanjing, China; 4 Department of General Surgery, the First Affiliated Hospital with Nanjing Medical University, 300 Guangzhou Road, 210029, Nanjing, China

## Abstract

The thyroid imaging reporting and data system (TIRADS) and Bethesda system for reporting thyroid cytopathology (BSRTC) have been used for interpretation of ultrasound and fine-needle aspiration cytology (FNAC) results of thyroid nodules. BRAF^V600E^ mutation analysis is a molecular tool in diagnosing thyroid carcinoma. Our objective was to compare the diagnostic value of these methods in differentiating high-risk thyroid nodules. Total 220 patients with high-risk thyroid nodules were recruited in this prospective study. They all underwent ultrasound, FNAC and BRAF^V600E^ mutation analysis. The sensitivity and specificity of TIRADS were 73.1% and 88.4%. BSRTC had higher specificity (97.7%) and similar sensitivity (77.6%) compared with TIRADS. The sensitivity and specificity of BRAF^V600E^ mutation (85.1%, 100%) were the highest. The combination of BSRTC and BRAF^V600E^ mutation analysis significantly increased the efficiency, with 97.8% sensitivity, 97.7% specificity. In patients with BSRTC I-III, the mutation rate of BRAF^V600E^ was 64.5% in nodules with TIRADS 4B compared with 8.4% in nodules with TIRADS 3 or 4A (*P* < 0.001). Our study indicated that combination of BSRTC and BRAF^V600E^ mutation analysis bears a great value in differentiating high-risk thyroid nodules. The TIRADS is useful in selecting high-risk patients for FNAB and patients with BSRTC I-III for BRAF^V600E^ mutation analysis.

Thyroid nodules are very common, with estimated prevalence ranging from 3% to 7% by palpation and 20% to 76% by ultrasound in the general population^1^. Although the majority of thyroid nodules are benign, differentiating malignancy from benign lesions is still the most challenging dilemma for clinicians. High-resolution ultrasound (HRUS) is recommended as the first line modality in the evaluation of thyroid nodules^1^. Solid composition, hypoechogenicity, microcalcification, irregular margin, taller-than-wide shape and increased blood flow are ultrasound (US) predictors for thyroid malignancy^2^. However, the sensitivities and specificities for these sonographic features vary greatly in different studies. The combination of several suspicious US features is more accurate than any single characteristic in predicting malignancy^3^,^4^. In 2009, Horvath *et al.*^5^ established a thyroid imaging reporting and data system (TIRADS) to stratify cancer risk based on 10 US patterns. A recent meta-analysis enrolled five studies with 7,753 thyroid nodules and showed that TIRADS had a pooled sensitivity and specificity of 0.75 and 0.69, respectively. However, due to different US equipment, criteria as well as the inevitable inter-observer variability among radiologists, there was a large range of sensitivity and specificity (0.57–0.96 and 0.43–0.94) with high heterogeneity^6^.

US-guided fine-needle aspiration biopsy (FNAB) is currently the essential triage approach for the preoperative evaluation of high-risk thyroid nodules. Ultrasound and clinical characteristics provided indications for FNAB such as high-risk history with suspicious US features like microcalcifications and irregular margins^1^. The Bethesda system for reporting thyroid cytopathology (BSRTC) was developed by American National Cancer Institute in 2009 to standardize the interpretation of the fine-needle aspiration cytology (FNAC) results^7^. The BSRTC is helpful in improving patient management and has been widely used. However, nondiagnostic and indeterminate results might bring dilemma to both patients and clinicians, the extent of which depends on the nodule composition, the skills of the operator and the experience of cytologist.

Recently, researchers attempted to seek molecular markers that may enhance the diagnostic value of FNAB. The thymine-to-adenine transversion at nucleotide position 1799 in exon 15 of the BRAF gene results in a valine-to-glutamate substitution at residue 600 (V600E), leading to constitutive activation of MAPK signaling downstream and the tumorigenesis of thyroid cells in the end^8^. Studies have shown that BRAF^V600E^ mutation is only present in papillary thyroid carcinoma (PTC) and PTC-derived anaplastic thyroid cancer. The mutation rate of BRAF^V600E^ ranges from 29% to 83% in PTC, with an average level of 45%^9^,^10^,^11^,^12^. It has been demonstrated that the combination of BRAF^V600E^ mutation analysis and cytology results can improve diagnostic performances of FNAB^13^,^14^.

In this study, we evaluated the diagnostic value of TIRADS, BSRTC and BRAF^V600E^ mutation analysis in differentiating high-risk thyroid nodules and explored the utilization of these methods for better diagnostic performance.

## Results

### Patients findings

A total of 220 patients (43 men and 177 women) with 220 thyroid nodules, including 86 benign nodules and 134 differentiated thyroid cancers (131 PTCs and 3 follicular thyroid cancers (FTCs)), were enrolled in this study. The average age was 44.9 ± 13.7 years old and the mean diameter of the nodules was 1.2 ± 0.7 cm. Detailed information on demographic and clinical features of the subjects were shown in Table 1. The mean age, size and FT4 level showed significant difference between benign and malignant groups (*P* < 0.05). While gender and FT3, TSH level showed no statistical difference between two groups (*P* > 0.05).

### Diagnostic value of TIRADS, BSRTC and BRAF^V600E^ mutation analysis

According to postoperative histopathology or FNAC and follow-up US, **t**he malignant rates of nodules categorized as TIRADS 3, 4A, 4B and 5 were 10.5%, 54.5%, 89.4% and 100%, respectively (Table 2). The receiver operating characteristic (ROC) curve demonstrated that the best cutoff of TIRADS was 4B. The sensitivity, specificity and AUC were 73.1%, 88.4% and 0.808, respectively (Table 3).

Of the 220 FNABs, 43 were BSRTC I (insufficient for cytology assessment), 12 of them (27.9%) were identified as PTCs by postoperative histopathology. Of the 38 BSRTC II (benign), 3 were demonstrated as PTCs after surgery. Of the 33 BSRTC III (AUS/FLUS), 15 nodules (45.5%) proved to be malignant. Of the 4 BSRTC IV (FN/SFN), 3 (75.0%) were validated as FTCs and 1 was regarded as follicular adenoma by histopathology. All 37 BSRTC VI (malignancy) and 64 of 65 BSRTC V (suspicious for malignancy) underwent surgery and proved to be PTCs histopathologically (Table 4). The remaining one nodule categorized as BSRTC V proved to be nodular goiter postoperatively. The ROC curve demonstrated that the best cutoff of BSRTC was IV. The sensitivity, specificity and AUC were 77.6%, 97.7% and 0.876, respectively (Table 3).

All the 220 specimens had definite genetic results. All the 114 nodules with BRAF^V600E^ mutation proved to be PTCs by postoperative histopathology. Among the 106 nodules negative for BRAF^V600E^ mutation, 17 PTCs, 3 FTCs and 25 benign lesions were validated after surgery, while the remaining 61 cases were diagnosed by followed-up FNAB or US. The BRAF^V600E^ mutation rates in BSRTC I to VI lesions were 23.3%, 7.9%, 42.4%, 0%, 84.6% and 86.5%, respectively (Table 4). Concerning BRAF^V600E^ mutation results, the AUC in detecting malignancy was 0.925, with a sensitivity of 85.1% and specificity of 100.0% (Table 3).

### Diagnostic value of combinations of different methods

The combination of TIRADS and BSRTC significantly increased the sensitivity (92.5%) compared to cytology alone, while the specificity (86.1%) and AUC (0.893) were not improved obviously. The combination of BSRTC and BRAF^V600E^ mutation analysis showed best AUC (0.977) and high sensitivity and specificity (97.8%, 97.7%). When three methods combined, the sensitivity increased to 98.5% with a decreased specificity to 86.1% (Table 3). Statistical analysis of ROCs showed that the AUC of BSRTC-BRAF^V600E^ mutation analysis was significantly higher as compared with each method alone (TIRADS *P* = 0.0001, BSRTC *P* < 0.0001, BRAF^V600E^ mutation *P* = 0.006) or the combination of three methods (*P* = 0.0003).

As shown in Table 4, among the 114 patients with BSRTC I-III, 30 (26.3%) were clearly diagnosed as PTCs histopathologically. The cancer risk in nodules with BRAF^V600E^ mutation was 100%, significantly higher than that with wild-type BRAF (3.4%, *P* < 0.0001). Moreover, in BSRTC I-III lesions, the cancer risk increased when the TIRADS classification was 4B or higher as compared with 3 or 4A (54.5% vs. 18.8%, *P* = 0.0583, 50.0% vs. 0%, *P* < 0.0008, 85.7% vs. 15.8%, *P* = 0.0003, respectively).

We then focused on the correlation of TIRADS classification and BRAF^V600E^ mutation in patients with BSRTC I-III, which was shown in Table 5. In nodules classified as TIRADS 3, 4A, 4B, the malignant rates were 3.8%, 22.6% and 67.7%, respectively. The BRAF^V600E^ mutation was identified in 64.5% (20/31) of nodules with TIRADS 4B and in 8.4% (7/83) of nodules with TIRADS 3 or 4A. The difference between BRAF^V600E^ mutation and TIRADS classification was statistically significant (*P* < 0.0001) (Table 5).

## Discussion

In this study, we evaluated the efficiency of TIRADS, BSRTC and BRAF^V600E^ mutation analysis in differentiating high-risk thyroid nodules in clinical practice. We found that BRAF^V600E^ mutation detection had the best sensitivity, specificity and accuracy among the three methods. The TIRADS applied to selecting patient for FNAB and BRAF^V600E^ mutation analysis. Both TIRADS and BRAF^V600E^ detection could increase the sensitivity and accuracy when combined with BSRTC. Of all the methods, combination of BSRTC and BRAF^V600E^ mutation detection reached the best diagnostic efficiency.

Ultrasound is the most sensitive modality available to detect thyroid lesions, which enable physicians to measure their dimensions, identify their structures, detect sonographic features suggestive of malignancy and select the high-risk lesions for FNAB. The TIRADS improved the diagnostic value of US and provided clinicians with more information to classify benign and malignant nodules. Similar to the results of Wei’s meta-analysis^6^, the present study demonstrated that the sensitivity and specificity of TIRADS reached 73.1% and 88.4%. However, the malignant rates in the nodules classified as TIRADS 3 and 4A (10.5%, 54.6%) were significantly higher than the ideal range (<5%, 5–10%) recommended by Horvath *et al.*^5^. This was probably due to the difference of radiologists’ experience, intra-observer variability, US criteria and devices, which may lead to misdiagnosis and improper management of some patients.

FNAC is currently the most reliable nonsurgical approach for the diagnosis of thyroid nodules. The formulation of BSRTC standardized the thyroid-reporting cytopathology^7^. Recently, a meta-analysis showed that the proportion of nondiagnostic samples accounted for 12.9%, and the rates of malignancy in BSRTC I-VI were 16.8%, 3.7%, 15.9%, 26.1%, 75.2% and 98.6%, respectively. In nodules that classified as BSRTC II and BSRTC IV-VI, the sensitivity, specificity and accuracy were 97%, 50.7% and 68.8% when based on histopathology results^15^. In the present study, the proportion of nondiagnostic samples reached 19.5%, which was much higher than the above level^15^, indicating that the requirements of specimens for BSRTC were high^16^ and our skill of FNAB to obtain adequate samples still needed to be improved. In addition, the proportion of AUS/FLUS in our study was 15.0%, higher than the recommended level (7%), but close to the reported results^15^,^17^. A recent study showed that the usage of FLUS varied substantially among pathologists and institutions, which was related with years of experience of pathologists and their training in cytopathology or not^18^. Moreover, the rates of malignancy in nodules that classified as BSRTC I-VI in this study were significantly higher than the recommended range, especially in the BSRTC I and III category (27.9% vs. 1–4%, 45.5% vs. 5–15%, respectively), revealing a relatively conservative attitude of the pathologist for interpreting the cytology. In BSRTC II and BSRTC IV-VI nodules, the sensitivity of cytology (97.2%) was close to the results of the meta-analysis, but the specificity (94.6%) was higher. When taken all 6 categories into consideration, the sensitivity fell to 77.6%, along with an increase in specificity (97.7%).

BRAF^V600E^ mutation analysis is a breakthrough in the molecular diagnosis of thyroid carcinoma in recent years^19^,^20^. It is the most common genetic alteration in thyroid cancer, occurring in about 45% of sporadic PTCs^21^. In present study, all the nodules with BRAF^V600E^ mutation were confirmed to be PTCs. The mutation rate of BRAF^V600E^ in PTCs in this study population was 85.1%, close to that in the Korea population^22^,^23^, but higher than the average level reported in the literatures. Furthermore, the sensitivity (85.1%) and specificity (100%) were also higher than the results of Jia’s meta-analysis (pooled sensitivity 59.3%; pooled specificity 99.0%)^24^. Areas and ethnic variations may be the main contributors to this difference^25^. In addition, different methods of gene mutation detection will also have impact on the results. The core technique used in this study was amplification refractory mutation system (ARMS), which is more sensitive and specific than the traditional methods like DNA sequencing^23^,^26^,^27^. The overall sensitivity and specificity of FNA-BRAF^V600E^ detection were much better than those of ultrasound or cytology, making it a powerful adjunct in stratifying high-risk thyroid nodules in China population.

In addition, we further explored the utilization of the combination of different methods. Of the 134 malignant cases validated by histopathology, 77 nodules were classified as positive for malignancy by both TIRADS and BSRTC; 27 nodules were positive only by TIRADS and 21 only by BSRTC. Combination of TIRADS and BSRTC could increase the sensitivity and AUC (92.5% and 0.893). Moreover, the BRAF^V600E^ mutation testing allowed to correct preoperative diagnosis in 27 patients diagnosed as BSRTC I-III, resulting in a significant increase in the sensitivity, which was agreed with previous reports^13^,^14^,^28^. The accuracy of cytology diagnosis depends on pathologists’ experience and sample quantity while BRAF^V600E^ mutation analysis only needs appropriate sample and standardized test. The combination of cytology and BRAF^V600E^ mutation analysis reached the best diagnostic performance, with 97.8% sensitivity and 97.7% specificity. A combination of US, FNAC and BRAF^V600E^ further increased the sensitivity at the cost of decreased specificity, which would limit its clinical application. Our results indicated that the TIRADS could be used as the preliminary evaluation method to select high-risk lesions for FNAB, while BSRTC and BRAF^V600E^ mutation analysis should be adopted to make the diagnosis.

In fact, since the high specificity and positive predictive value of BSRTC, patients with nodules diagnosed as BSRTC IV-VI should all be recommended for surgery^7^. Additional BRAF^V600E^ mutation testing has little value in improving the diagnostic efficiency of FNAB in these patients. However, of the 30 cases that were classified as BSRTC I-III but proved to be carcinomas by histopathology, 27 were positive for BRAF^V600E^ mutation, indicating the vital value of BRAF mutation analysis in lesions with BSRTC I-III. Moreover, we found that the increased level of TIRADS classification was significantly associated with the rising mutation rate of BRAF^V600E^ in each BSRTC category, demonstrating that the TIRADS could be used to select patients for molecular analysis. Our results show the great value to detect BRAF^V600E^ mutation if the nodule is TIRADS 4B along with BSRTC I-III, partly in line with literatures^29^.

The limitations of our study should also be addressed. Firstly, not all thyroid nodules underwent surgery in this study. Some final diagnosis were based on cytology and follow-up US, which may cause some false negative results and overestimated sensitivity of BRAF^V600E^ mutation analysis. Secondly, due to the relatively high percentage of BSRTC I and III in this study, the extra diagnostic value of BRAF^V600E^ mutation analysis might be exaggerated in some degree. Moreover, our study focused on high-risk thyroid nodules by ultrasound and the frequency of malignancy was higher than expected in a general population of patients with thyroid nodules, so the selecting bias was unavoidable. Since sensitivity, specificity also depend on the expected frequency of the investigated outcome, our results need to be confirmed with a prospective study on a nonsurgical population.

In conclusion, the diagnostic performance of BRAF^V600E^ mutation analysis is the best among three methods. The combination of BSRTC and BRAF^V600E^ mutation analysis is the most reliable and efficient method for diagnosing thyroid cancer, especially PTCs. In patients with BSRTC I-III, the TIRADS is useful to select patients for FNA-BRAF^V600E^ mutation analysis and modify the diagnosis and clinical management. Further studies will still need to be done to verify our results, standardize the usage of BRAF^V600E^ mutation analysis and improve the preoperative diagnostic level of thyroid nodules.

## Methods

### Subjects

**Figure 1:**
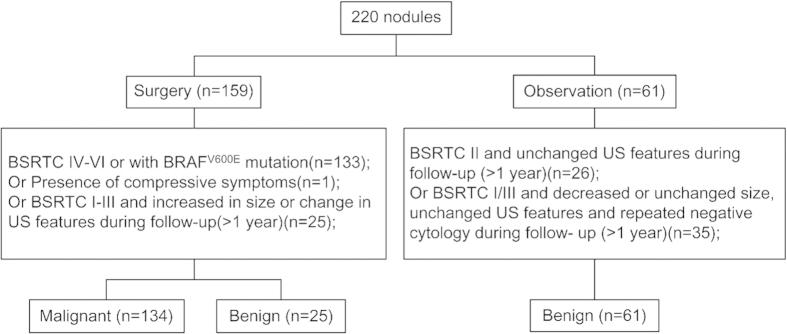
Diagram of the study group.

This was a prospective study. Consecutive patients with high risk thyroid nodules after US evaluation who need further FNAB examination were selected in our hospital from January to April 2014. Inclusion criteria: (1) US evaluation as TIRADS 4–6, or (2) TIRADS 3 that meet at least one of the following criteria: the nodule grows during follow-up (more than a 50% change in volume or a 20% increase in at least two nodule dimensions with a minimal increase of 2 mm in solid nodules or in the solid portion of mixed cystic-solid nodules), patients with higher risk of malignancy like those exposed to previous radiation to the neck, those with family history of thyroid cancer^5^. FNAB with an additional BRAF^V600E^ mutation analysis was performed. If a patient had multiple nodules, the one showed highest risk of malignancy or largest size was further assessed. BSRTC was applied to interpret the FNAC results. Total 233 patients with 233 nodules were included preliminarily. One with cytology BSRTC V was excluded because he refused surgery. Twelve with cytology BSRTC I (n = 4), BSRTC II (n = 3) and BSRTC III (n = 5) were excluded due to drop-out. Finally, a total of 220 patients with 220 thyroid nodules were enrolled, of them 159 patients underwent surgery while 61 patients received follow-up (Fig. 1). For the observation group, patients with BSRTC II cytology underwent US examination every six months, while patients with BSRTC I/III cytology underwent repeated FNAB and ultrasound examination after three months and continued follow-up by US every six months if repeated cytology was still negative. By April 2015, the duration of follow-up was more than one year for every patient. All the 134 malignant lesions were validated by surgery, while 29.1% of the benign lesions were diagnosed based on histopathology and 70.9% based on observation (Fig. 1). Informed consent was obtained from all patients and the study was performed in accordance with the ethical guidelines of the Helsinki Declaration and approved by the local ethics review committee (2012-SR-057).

### Clinical profiles

Patient characteristics including age, sex, related hormone level (FT3, FT4, TSH), and diameter of each thyroid nodule were recorded. The differences of these clinical findings between benign and malignant lesions were compared.

### TIRADS classification

A Mylab Twice ultrasound unit (The Esaote Group, Genova, Italy) equipped with a commercially available 4–13 MHz linear-array transducer were utilized to perform careful US examination of the thyroid gland and neck region. According to the criteria proposed by Horvath *et al.*^5^, all nodules were classified into four categories: TI-RADS 3: Hyper, iso, or hypoechoic, partially encapsulated nodule with peripheral vascularization, in Hashimoto’s thyroiditis; TI-RADS 4A: Solid or mixed hyper, iso, or hypoechoic nodule, with a thin capsule. Hypoechoic lesion with ill-defined borders, without calcifications. Hyper, iso, or hypoechoic, hypervascularized, encapsulated nodule with a thick capsule, containing calcifications (coarse or microcalcifications); TI-RADS 4B: Hypoechoic, nonencapsulated nodule, with irregular shape and margins, penetrating vessels, with or without calcifications; TI-RADS 5: Iso or hypoechoic, nonencapsulated nodule with multiple peripheral microcalcifications and hypervascularization. One radiologist with working experience of four years did all the classifications.

### FNAB

Pre-operative FNABs were performed under ultrasound guidance with a 25-gauge needle attached to a 5-ml syringe. Each lesion was aspirated three to four passes in different directions. The aspirates of 2–3 passes were expelled on glass slides, smeared, and placed immediately in 95% alcohol and then were sent for cytology analysis by one experienced cytopathologist. The remaining one pass of material was rinsed in 180 uL cytolysis liquid and used for genetic analysis. The cytopathologist was not on-site during the biopsy. The criterion for an adequate smear was the presence of six groups of cells with more than 10 cells per group. Based on the BSRTC, cytology results were categorized as I-VI: nondiagnostic, benign, atypia undetermined significance/follicular lesion of undetermined significance (AUS/FLUS), follicular neoplasm or suspicious for a follicular neoplasm (FN/SFN), suspicious for malignancy and malignancy^7^.

### DNA isolation and BRAF^V600E^ detection

BRAF^V600E^ mutation analysis was performed at the laboratory of the section of pathology of our hospital. DNA extraction was successfully completed in all samples following the manufacturer’s instructions with a commercially available kit (ADx-ARMS, AmoyDX, Xiamen, China). The quantity of isolated DNA was assessed by using a NanoDrop2000 spectrophotometer (Thermo, L.A., USA). All samples in this study were adequate for genetic detection. The samples were analyzed by applying the amplification refractory mutation system (ARMS) technique. ARMS-PCR is a highly sensitive method employed for genotyping specific single nucleotide mutations. Briefly, each PCR reaction mixture contained 5 μl of extracted DNA and other chemicals available in a kit (ADx-ARMS, AmoyDX, Xiamen, China) containing oligonucleotide primers, TaqDNA polymerase, oligonucleotide probes, nucleotides, and buffers. The PCR reaction was carried out on a qRT-PCR machine (ABI7900, USA) with an initial denaturation step at 94 °C for 5 minutes, then 15 annealing cycles at 95 °C for 25 seconds, 64 °C for 20 seconds, and 72 °C for 20 seconds, followed by 31 extension cycles at 93 °C for 25 seconds, 60 °C for 35 seconds, 72 °C for 20 seconds. Fluorescence increased geometrically corresponding to the exponential increase of the PCR products, which was used to determine threshold cycle (*C*_T_). If the *C*_T_ value was less than 28, it was considered as positive, otherwise as negative^30^.

### Statistical analysis

Statistical analysis was performed using SPSS 13.0 software (SPSS Inc., Chicago, USA). All quantitative values were expressed as means ± SD. Differences in the distribution of categorical variables between groups were evaluated by the 2-tailed Chi-square (χ^2^) test or Fisher exact test. The best diagnostic cutoff value of TIRADS and BSRTC were determined by ROC curves. When two diagnostic tests combined, a nodule was considered positive when either test was positive. According to final diagnosis, the sensitivity, specificity, positive predictive value (PPV) and negative predictive value (NPV) were calculated. The ROC curves were graphed and the areas under the curves (AUCs) were calculated to compare the diagnostic efficiency of these methods. *P* < 0.05 was considered significant in all tests.

## Additional Information

**How to cite this article**: Zhang, Y.-z. *et al.* Value of TIRADS, BSRTC and FNA-BRAF^V600E^ mutation analysis in differentiating high-risk thyroid nodules. *Sci. Rep.* **5**, 16927; doi: 10.1038/srep16927 (2015).

## Figures and Tables

**Table 1:** Clinical features of the study population.

Features		Benign	Malignant	*P* value
Sex	Male	15	28	0.60
	Female	71	106	
Age (year)		48.78 ± 13.89	42.41 ± 12.94	0.002
Diameter (cm)		1.66 ± 0.90	1.06 ± 0.58	0.000
FT3 (pmol/L)		4.73 ± 0.63	4.90 ± 1.27	0.94
FT4 (pmol/L)		16.06 ± 2.97	17.52 ± 4.75	0.04
TSH (mIU/L)		2.43 ± 1.89	3.23 ± 9.75	0.62

Data are presented as mean (SEM).

**Table 2:** Correlation of TIRADS classifications and final diagnosis.

TIRADSclassification	n	Surgery (%)	Benign	Malignant	Malignant Rate(95% CI)
3	57	31.6	51	6	10.5 (4.0–21.5)
4A	55	67.3	25	30	54.5 (40.6–68.0)
4B	94	95.7	10	84	89.4 (81.3–94.8)
5	14	100	0	14	100 (76.8–100.0)
Total	220	72.3	86	134	60.9 (54.1–67.4)

Abbreviations: CI, confidence interval.

**Table 3:** Comparison of the diagnostic value of TIRADS, BSRTC and BRAF^V600E^ mutation Analysis.

Statistics	TIRADS	BSRTC	BRAF^V600E^mutation	TIRADS + BSRTC	BSRTC + BRAF^V600E^mutation	TIRADS + BSRTC + BRAF^V600E^mutation
Sensitivity (95% CI)	73.1 (64.8–80.4)	77.6 (69.6–84.4)	85.1 (77.9–90.6)	92.5 (86.7–96.4)	97.8 (93.6–99.5)	98.5 (94.7–99.8)
Specificity (95% CI)	88.4 (79.7–94.3)	97.7 (91.9–99.7)	100.0 (95.8–100.0)	86.1 (76.9–92.6)	97.7 (91.9–99.7)	86.1 (76.9–92.6)
PPV (95% CI)	90.7 (83.6–95.5)	98.1 (93.4–99.8)	100.0 (96.8–100.0)	91.2 (85.1–95.4)	98.5 (94.7–99.8)	91.7 (85.9–95.6)
NPV (95% CI)	67.9 (58.3–76.4)	73.7 (64.6–81.5)	81.1 (72.3–88.1)	88.1 (79.1–94.2)	96.6 (90.2–99.3)	97.4 (90.7–99.7)
AUC (95% CI)	0.808 (0.749–0.857)	0.876 (0.826–0.917)	0.925 (0.882–0.956)	0.893 (0.844–0.930)	0.977 (0.948–0.993)	0.923 (0.879–0.954)

Abbreviations: PPV, positive predictive value; NPV, negative predictive value; AUC, area under curve; CI, confidence interval.

**Table 4:** Correlation of BSRTC categories with BRAF^V600E^ mutation and final diagnosis.

BSRTC categories	n (%)	Surgery (%)	BRAF^V600E^mutation (%)	Malignant	Malignant Rate(95% CI)
I (Nondiagnostic)	43 (19.5)	20 (46.5)	10 (23.3)	12	27.9 (13.3–43.7)
II (Benign)	38 (17.3)	12 (31.6)	3 (7.9)	3	7.9 (1.7–21.4)
III (AUS/FLUS)	33 (15.0)	21 (63.6)	14 (42.4)	15	45.5 (28.1–63.7)
IV (FN/SFN)	4 (1.8)	4 (100.0)	0 (0.0)	3	75.0 (19.4–99.4)
V (Suspicious for malignancy)	65 (29.5)	65 (100.0)	55 (84.6)	64	98.5 (91.7–100.0)
VI (Malignancy)	37 (16.8)	37 (100.0)	32 (86.5)	37	100.0 (90.5–100.0)

Abbreviations: AUS/FLUS, atypia of undetermined significance or follicular lesion of undetermined significance; FN/SFN, follicular neoplasm or suspicious for a follicular neoplasm; CI, confidence interval.

**Table 5:** Correlation of TIRADS classification with BRAF^V600E^ mutation and final diagnosis in BSRTC I-III categories.

TIRADS	BSRTC I		BSRTC II		BSRTC III		BSRTC I-III		
	n	BRAF^V600E^mutation (%)	n	BRAF^V600E^mutation (%)	n	BRAF^V600E^mutation (%)	n	BRAF^V600E^mutation (%)	Malignancy (%)
3	19	2 (10.5)	24	0	9	0	52	2 (3.8)	2 (3.8)
4A	13	2 (15.3)	8	0	10	3 (30.0)	31	5 (16.1)	7 (22.6)
4B	11	6 (54.5)	6	3 (50.0)	14	11 (78.6)	31	20 (64.5)	21 (67.7)
Total	43	10 (23.3)	38	3 (7.9)	33	14 (42.4)	114	27 (23.7)	30 (26.3)
